# Supplementary material for: Deletion of wfs1 Impairs Oligodendrocyte Precursor Cells Dorsal Distribution and Myelination Through the wfs1-hmgcs1 Axis in Zebrafish
Source: Biology (Basel). 2026 Aug 16;15(16):1402. doi: 10.3390/biology15161402 (PMC13510151; doi:10.3390/biology15161402)
Supplement: Supplementary file 1 [file biology-15-01402-s001.zip › File S1-Transcriptome sequencing data and differentially expressed genes (DEGs) in wfs1-- zebrafish at 5 dpf/2.KEGG/wfs1KOvsWT/ALL/src/dre04070.html]

dre04070


- dre:192322

- Up regulated genes

336121(1.42904963424916)

- dre:100000252

- Up regulated genes

100000252\_3(1.94434154854557) 100000252(1.09163460881879)

- Down regulated genes

100000252\_1(-1.7718817168434)

- dre:100000252

- Up regulated genes

100000252\_3(1.94434154854557) 100000252(1.09163460881879)

- Down regulated genes

100000252\_1(-1.7718817168434)
- dre:571356

- Up regulated genes

571356(1.35534919146634) 571356\_2(3.19114319937523)

- Down regulated genes

571356\_1(-1.36763102903653)

- dre:100002015

- Up regulated genes

100002015\_1(2.93882889369414)

- dre:724010

- Up regulated genes

724010\_1(10.241655134474)

- Down regulated genes

724010(-6.84228486467569)

- dre:100000252

- Up regulated genes

100000252\_3(1.94434154854557) 100000252(1.09163460881879)

- Down regulated genes

100000252\_1(-1.7718817168434)
- dre:571356

- Up regulated genes

571356(1.35534919146634) 571356\_2(3.19114319937523)

- Down regulated genes

571356\_1(-1.36763102903653)

- dre:337236

- Up regulated genes

337236\_2(8.533525538938) 337236(6.56374522648181)

- Down regulated genes

337236\_1(-2.86219060000093)

- dre:368717

- Up regulated genes

368717(4.12704824157962)

- Down regulated genes

368717\_1(-1.74128930765284)

- dre:678558

- Up regulated genes

678558\_1(6.03678629308862)
- dre:559198

- Up regulated genes

559198(1.99669187801435)

- Down regulated genes

559198\_1(-1.12263541156314)

- dre:566642

- Up regulated genes

566642\_1(5.98783405066651)

- Down regulated genes

566642(-2.64539739093262)

- dre:678558

- Up regulated genes

678558\_1(6.03678629308862)
- dre:559198

- Up regulated genes

559198(1.99669187801435)

- Down regulated genes

559198\_1(-1.12263541156314)

- dre:559938

- Up regulated genes

559938\_1(6.88721649670372)

- Down regulated genes

559938(-1.00400083336952)

- dre:559938

- Up regulated genes

559938\_1(6.88721649670372)

- Down regulated genes

559938(-1.00400083336952)

- dre:100000252

- Up regulated genes

100000252\_3(1.94434154854557) 100000252(1.09163460881879)

- Down regulated genes

100000252\_1(-1.7718817168434)

- dre:100006675

- Up regulated genes

100006675(1.63301233543285)
- dre:497384

- Down regulated genes

497384\_1(-2.05267237809407)

- dre:337236

- Up regulated genes

337236\_2(8.533525538938) 337236(6.56374522648181)

- Down regulated genes

337236\_1(-2.86219060000093)

Close
